# Chronic exposure to ambient particulate matter induces gut microbial dysbiosis in a rat COPD model

**DOI:** 10.1186/s12931-020-01529-3

**Published:** 2020-10-19

**Authors:** Naijian Li, Zhaowei Yang, Baoling Liao, Tianhui Pan, Jinding Pu, Binwei Hao, Zhenli Fu, Weitao Cao, Yuming Zhou, Fang He, Bing Li, Pixin Ran

**Affiliations:** 1 Department of Allergy and Clinical Immunology, State Key Laboratory of Respiratory Disease, National Clinical Research Center for Respiratory Disease, Guangzhou Institute of Respiratory Health, The First Affiliated Hospital of Guangzhou Medical University, Guangzhou, 510000 P.R. China; 2 Nanhai Hospital Southern Medical University, Foshan, 528000 P.R. China; 3 State Key Laboratory of Respiratory Disease, National Clinical Research Center for Respiratory Disease, Guangzhou Institute of Respiratory Health, The First Affiliated Hospital of Guangzhou Medical University, Guangzhou, 510000 P.R. China; 4 Department of Respiratory Medicine, The Third Affiliated Hospital of Guangzhou Medical University, Guangzhou, 510000 P.R. China; 5 The School of Basic Medicine, Guangzhou Medical University, Guangzhou, 510000 P.R. China; 6 The GMU-GIBH Joint School of Life Sciences, Guangzhou Medical University, Guangzhou, 510000 P.R. China

**Keywords:** Biomass fuel, Motor vehicle exhaust, COPD, Gut microbiome

## Abstract

**Background:**

The role of the microbiota in the pathogenesis of chronic obstructive pulmonary disease (COPD) following exposure to ambient particulate matter (PM) is largely unknown.

**Methods:**

Fifty-four male Sprague-Dawley rats were exposed to clean air, biomass fuel (BMF), or motor vehicle exhaust (MVE) for 4, 12, and 24 weeks. We performed pulmonary inflammation evaluation, morphometric measurements, and lung function analysis in rat lung at three different times points during exposure. Lung and gut microbial composition was assessed by 16S rRNA pyrosequencing. Serum lipopolysaccharide levels were measured and short-chain fatty acids in colon contents were quantified.

**Results:**

After a 24-week PM exposure, rats exhibited pulmonary inflammation and pathological changes characteristic of COPD. The control and PM exposure (BMF and MVE) groups showed similar microbial diversity and composition in rat lung. However, the gut microbiota after 24 weeks PM exposure was characterized by decreased microbial richness and diversity, distinct overall microbial composition, lower levels of short-chain fatty acids, and higher serum lipopolysaccharide.

**Conclusion:**

Chronic exposure to ambient particulate matter induces gut microbial dysbiosis and metabolite shifts in a rat model of chronic obstructive pulmonary disease.

## Introduction

Air pollution is a primary environmental cause of chronic respiratory diseases [1, 2]. Industrial activities, vehicular emissions, and household biomass combustion are major sources of ambient particulate matter (PM) [2]. Incomplete combustion of biomass fuel and living in proximity to traffic have been associated with a high prevalence of chronic obstructive pulmonary disease (COPD) [3, 4], while reductions in ambient PM have been shown to decrease the risk of COPD [5]. Therefore, it is important to elucidate the mechanism of ambient PM-induced COPD.

In a previous study, we established a rat COPD model via chronic exposure to biomass fuel (BMF) and motor vehicle exhaust (MVE), which can be used to investigate COPD in non-smokers [6]. We found that both BMF and MVE chronic exposure induced airway cells to secrete cytokines that develop pronounced COPD in rats, but its mechanisms are largely unknown. Over the past few years, research of PM has mostly focused on the inflammation response to inhalation, but emerging evidence has suggested that PM exposure may also cause microbial dysbiosis [7, 8]. Moreover, recent studies also have linked changes in the microbiome to COPD, the role of the microbiome in the development of COPD have drawn considerable attention [9, 10].

Although it is clear that chronic exposure to PM can induce COPD-like changes in rat lung, the effects of the microbiota on COPD following chronic exposure to ambient PM are largely unknown. We hypothesized that chronic exposure to ambient particulate matter induces gut and lung microbial dysbiosis in a rat COPD model. To test our hypothesis, we established a rat COPD model using exposures to BMF and MVE. Rats were exposed for 4, 12, and 24 weeks, and changes in pulmonary histopathology, microbial composition, and microbial metabolites (lipopolysaccharide [LPS] and short-chain fatty acids [SCFAs]) were measured.

## Methods

### Animals

Male Sprague-Dawley rats (7–9 weeks in age) were purchased from Guangzhou University of Chinese Medicine (Guangzhou, China). The Animal Medical Center of Guangzhou Medical University reviewed and approved all experiments (identification number: GY2019–009). A total of 54 rats were randomly divided into three groups: control, BMF, and MVE (*n* = 6 per group) for three exposure durations (4, 12, and 24 weeks). All rats were kept in a specific pathogen-free room and were housed three to a cage. Except the time of the PM exposure, the animal facility conditions of the BMF and MVE groups are the same as those of the control group. The animal facility maintained temperature and relative humidity at 23 ± 2 °C and 40–70%, respectively. Lighting was artificial with a sequence of 12 h light (06:00–18:00) and 12 h dark. Commercially available rodent food pellets and water were provided ad libitum. Rats were weighed every 2 weeks throughout the study. Corncob bedding and cage are replaced every 3 days. The rats were observed for any sign of illness a minimum of twice daily.

### PM exposure system and characterization of the test atmosphere

**Fig. 1:**
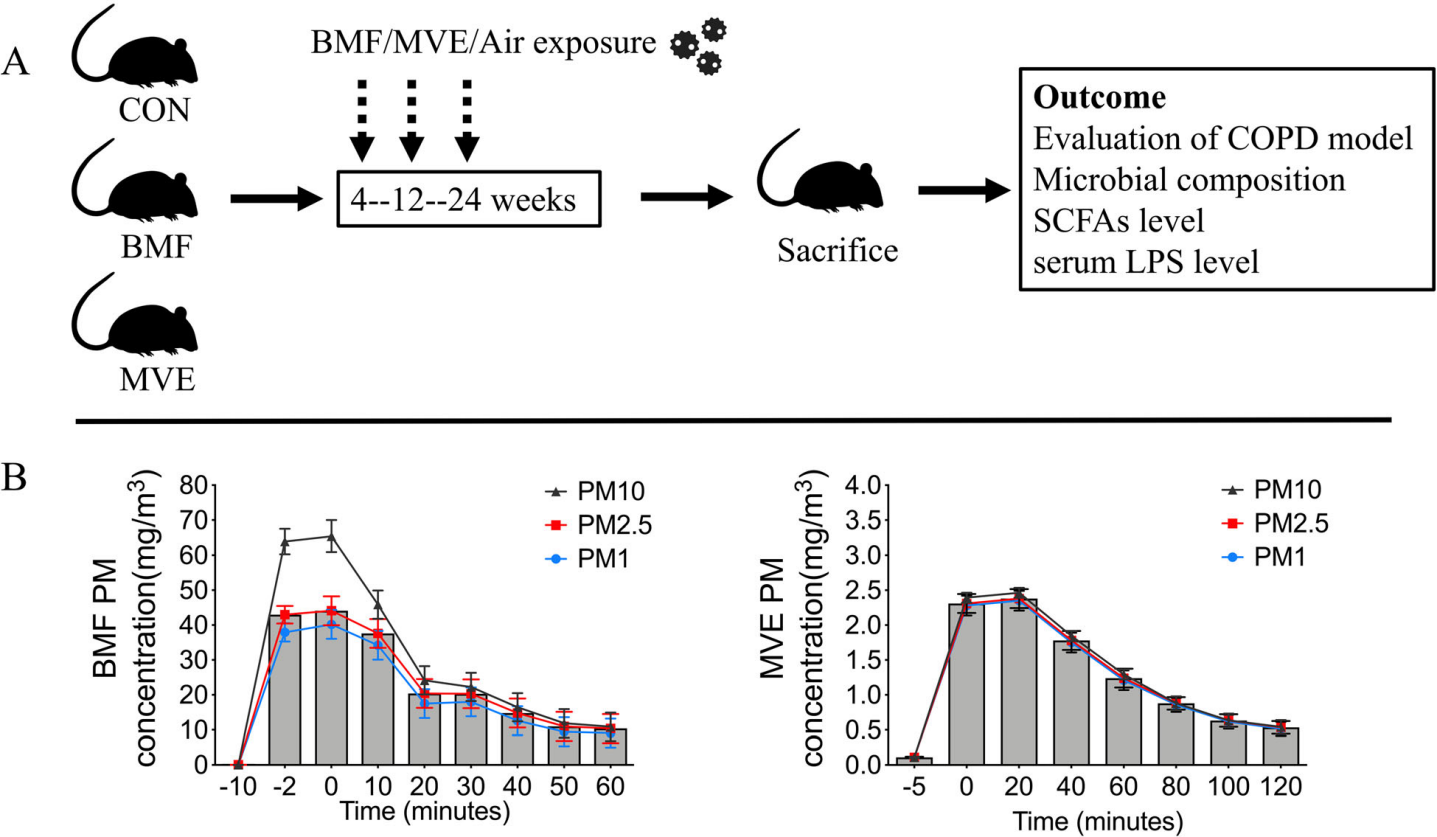
Schematic overview of the study workflow. **a** A total of 54 rats were randomly divided into three groups (control [CON], biomass fuel [BMF], and motor vehicle exhaust [MVE]; *n* = 6 per group) and exposed for 4, 12, or 24 weeks. Lung tissue was assessed histologically and gut microbial composition was assessed by 16S rRNA pyrosequencing. Serum lipopolysaccharide (LPS) levels were measured and short-chain fatty acids (SCFAs) in colon contents were quantified. **b** Particulate matter (PM) concentrations and particle size distributions during exposure. Rats exposed to biomass fuel (BMF) inhaled higher concentrations of PM with a diameter ≤ 10, 2.5, and 1 μm (PM_10_, PM_2.5_, and PM_1_) than rats exposed to motor vehicle exhaust (MVE). Boxes and the inside line represent the mean ± SD for PM_2.5_

Figure 1a depicts the design of the study. Rats were exposed to PM as described previously [6]. Briefly, all animals were exposed in whole-body inhalation chambers for 4 h/day, 5 days/week either 4, 12, or 24 weeks. PM mass concentrations, particle size distributions, and gas concentrations were monitored each day of exposure. DustTrak II aerosol monitors (model 8530, TSI, Shoreview, MN, USA) were used to monitored PM mass concentrations and particle size distributions. Testo 340 portable flue gas analyzers (Testo, Lenzkirch, Germany) were used to monitored gas concentrations (O_2_, carbon monoxide, nitrogen oxides, and sulfur dioxide) in the exposure rooms. The control group was exposed to clean air.

#### Exposure to BMF

As Chinese fir (*Cunninghamia lanceolata*) is the major indigenous tree species that occupies approximately 25% of plantations in subtropical areas of southern China [11], we used Chinese fir sawdust (40 g/per exposure), as a representative, to produce BMF smoke, which was sent into the animal exposure room through a piston pump (5 L/min). Rats were exposed to BMF smoke for four 1-h periods, 5 days per week. The test atmosphere was measured during the first hour.

#### Exposure to MVE

Previous studies indicated that exposure to higher traffic-related air pollutants was strongly associated with increased COPD prevalence [4]. Therefore, the gasoline-powered motorcycle was used as a source of MVE to stimulate a real-world pollution. MVE was produced by a Wuyang model WY48QT-2, 1.6-Kw, 125-cm^3^, one-cylinder, four-cycle, gasoline-powered motorcycle (Guangzhou, China). Premium low-sulfur gasoline (< 150 ppm; Petro Inc., El Paso, TX, USA) was used to produce MVE. Prior to the exposure session, the motorcycle engine was operated in an idle state for 2 min to produce sufficient MVE. Rats were exposed for two 2-h periods, 5 days per week. The test atmosphere was measured during the first 2-h interval.

Ambient BMF and MVE samples were collected throughout the duration of exposure to determinate the mass concentration and composition of atmospheric aerosol. Concentrations of organic carbon, elemental carbon, polycyclic aromatic hydrocarbons, and metals were further measured at the Guangzhou Institute of Chemistry, Chinese Academy of Sciences (Guangzhou, China) according to previous studies [12, 13].

### Measurement of lung function

Spirometry data were obtained as previously described using a Forced Pulmonary Maneuver System (Buxco Research Systems, Wilmington, NC, USA) [6]. Rat were sedated with 3% pentobarbital (1 mL/Kg), tracheostomized and intubated, then placed supine in the body chamber and connected to the system. According to the procedures, the FRC (functional residual capacity), FEV20 (forced expiratory volume in 20 s), FEV100 (forced expiratory volume in 100 s) and PEF (peak expiratory flow) were measure. At least three acceptable maneuvers for each test of every mice were conducted to obtain a reliable mean spirometry data.

### Sample preparation

Rats were sacrificed by CO_2_ after 4, 12, and 24 weeks of exposure (on days 29, 85, and 169, respectively). Blood samples were collected from the heart and centrifuged at 1700×*g* for 15 min at 4 °C. Serum was stored at − 80 °C. Proximal colon contents were harvested using sterile instruments for each individual animal and site. Fresh proximal colon contents samples were snap-frozen in liquid nitrogen then stored at − 80 °C for microbial and SCFA analysis.

### Bronchoalveolar lavage fluid differential cell count

Bronchoalveolar lavage fluid (BALF) was collected as previously reported [14]. Cells were isolated by centrifugation at 300×*g* for 10 min at 4 °C and stained with Diff-Quik stain (Baso Diagnostics, Zhuhai, China). Differential cell counts were assessed from 400 cells counted on each slide.

### Lung morphometric analysis

As described previously [6], lung tissues were fixed with 4% paraformaldehyde solution and embedded in paraffin using standard methods. Sectioning and staining were performed by the Pathology Center of the First Affiliated Hospital of Guangzhou Medical University (Guangzhou, China). All slides were scanned and analyzed using an image analyzer platform (Leica, Wetzlar, Germany). Alveolar enlargement and destruction, and the bronchial wall thickness was calculated as describe previous [15].

### Serum levels of lipopolysaccharide and total BALF protein assay

Serum levels of LPS were measured using a commercial chromogenic end-point TAL kit (Xiamen Bioendo Technology Co., Ltd., Xiamen, China). All procedures were performed according to the manufacturer’s instructions. The total protein in the BALF determined by Bicinchoninic Acid (BCA) method using a commercial BCA Protein Assay Kit (Thermo Fisher Scientific, Waltham, USA). The concentration of endotoxin from DMSO-extract of particulate matter were performed at the Kingmed Diagnostics Center (Guangzhou, China). All procedures were performed according to the Pharmacopoeia of the China (2015 edition) volume IV.

### Microbiota analyses

DNA was isolated from colon contents and BALF samples using a Qiagen QIAamp® DNA extraction kit (Qiagen, Hilden, Germany) according to the manufacturer’s recommendations. 16S rRNA gene amplification, in vitro transcription, and labeling and hybridization were performed following the Illumina 16S Metagenomic Sequencing Library Preparation guide [16]. We used a MiSeq rRNA amplicon sequencing protocol to PCR-amplify the V3–V4 variable regions (amplicon size expected: approximately 460 bp). 16S amplicon PCR forward primer was 5′-(TCG TCG GCA GCG TCA GAT GTG TAT AAG AGA CAG CCT ACG GGN GGC WGC AG)-3′ and 16S amplicon PCR reverse primer was 5′-(GTC TCG TGG GCT CGG AGA TGT GTA TAA GAG ACA GGA CTA CHV GGG TAT CTA ATC C)-3′ [17]. All samples were paired-end sequenced on an Illumina MiSeq PE250 platform (San Diego, CA, USA) by the RiboBio Genome Center (Guangzhou, China). 16S rRNA gene sequence analysis, including raw sequence filtering and taxonomic classification, was performed as described previously [18]. The bar diagrams of alpha diversity indices and relative abundance were drawn using GraphPad Prism 8 software (La Jolla, CA, USA).

### Quantification of SCFAs in colon contents

Seven SCFAs (acetic, propionic, butyric, isobutyric, valeric, isovaleric, and caproic acids) in the proximal colon were measured by high-performance gas chromatography-mass spectrometry (Agilent 6890 N; Agilent Technologies, Santa Clara, CA, USA) according to the manufacturer’s recommendations. Briefly, a total of 100 mg of the colon contents were homogenized by ultrasonication in 600-μL reactions containing 100 μL of phosphoric acid (15%), 100 μL of 4-methylvaleric acid (250 μg/mL), and 400 μL of diethyl ether. The mixtures were vortexed and centrifuged until a clear supernatant was obtained. The sample was subjected to a high-performance liquid chromatography column (Agilent Technologies) for analysis of SCFAs.

### Statistical analysis

Data are reported as mean ± standard deviation (SD). Comparisons were performed using ANOVA and *p* values were corrected for multiple testing with the Bonferroni method. Statistical analysis was performed using SPSS version 24 (IBM SPSS, Armonk, NY, USA). Correlations between serum LPS levels and the pulmonary mean linear intercept (MLI) were assessed using Spearman’s rank correlation. *p* < 0.05 was considered significant.

## Results

### Particle size and gas concentrations during PM exposure

Particle size distributions and gas concentrations were measured during PM exposure. Figure 1b and supplementary Table 1 show the concentrations of PM with a diameter ≤ 10, 2.5, and 1 μm (PM_10_, PM_2.5_, and PM_1_) during BMF and MVE exposures. Average concentrations of PM_10_, PM_2.5_, and PM_1_ in the BMF exposure were 25.87 ± 2.99 mg/m^3^, 21.91 ± 1.84 mg/m^3^, and 19.60 ± 1.76 mg/m^3^, respectively. In the MVE exposure, average concentrations were 1.86 ± 0.20 mg/m^3^, 1.85 ± 0.24 mg/m^3^, and 1.82 ± 0.27 mg/m^3^, respectively. BMF exposure caused notably higher particulate emissions. Health impact assessments of PM considering not only mass concentration but also the composition of atmospheric aerosol. Supplementary Table 1 lists the mean ± SD concentrations of O_2_, carbon monoxide, nitrogen oxides, and sulfur dioxide during exposure. Supplementary Tables 2, 3 and 4 list the elemental composition of the particulates. The exposure concentrations of BMF and MVE reported here are based on our previous research on indoor air pollution in rural areas of China [4, 5, 19], and with those online reporting of the heavy haze conditions [20]. In contrast with our previous study [6], we improved the ventilation system and burning process to reduce the concentration of nitrogen oxides during the exposure, which is more in line with the real-world exposure.

In order to evaluate the concentration of LPS attached to particulate matter, we tested the concentration of endotoxin from DMSO-extract of particulate matter, which collected from BMF exposure room, MVE exposure room and the clean air. The endotoxin levels from BMF exposure room, MVE exposure room and the clean air were 12.0 EU/ml, 10.0 EU/ml and 12.5 EU/ml, respectively (Supplementary Table 5). Our data suggest that there is no significant difference in endotoxin levels from the three group during the exposure.

### Evaluation of COPD model

**Fig. 2:**
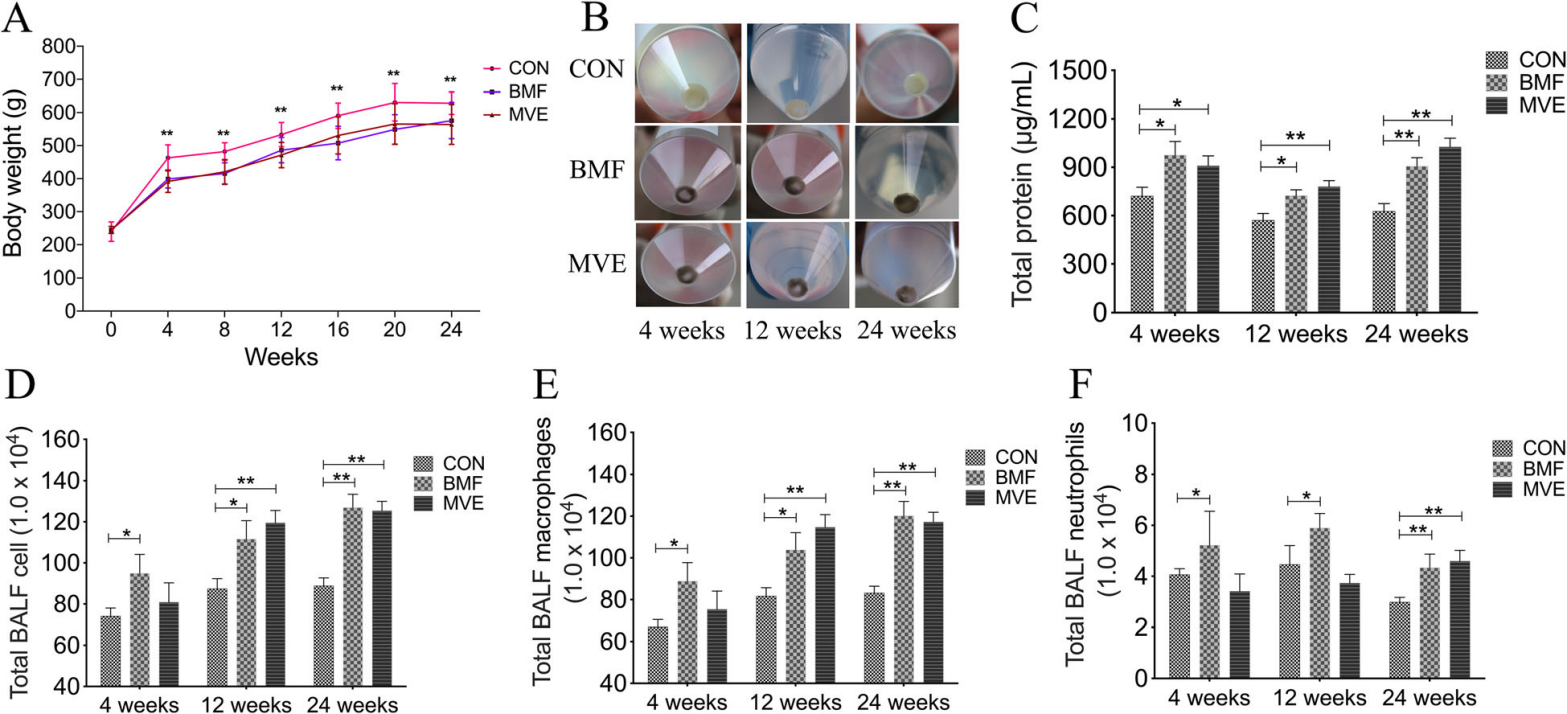
Exposure to biomass fuel (BMF) and motor vehicle exhaust (MVE) induces pulmonary inflammation in rats. **a** Effects of BMF and MVE exposure on body weight. **b** Particle sediments were observed in bronchoalveolar lavage fluid in the BMF and MVE groups during the exposure period (*n* = 6). **c** The levels of total protein in BALF. **d** BALF total leukocyte counts. **e**, **f** BALF alveolar macrophages counts and neutrophil counts. *n* = 6; *p < 0.05, **p < 0.01. CON, control group

Exposure to PM affected body weight and induced pulmonary inflammation. Rats continued to gain weight during PM exposure, with a total weight gain of 162.3% (controls), 135.2% (BMF group), and 130.4% (MVE group). The BMF and MVE groups maintained a steady rate of weight gain throughout the exposure period, but the rate was slower than the rate of weight gain in the controls after 4 weeks (*p* < 0.01; Fig. 2a). The rats exposed to BMF or MVE had more sediment particulate matter in BALF as early as 4 weeks into the exposure period (Fig. 2b). The levels of total protein in BALF were both higher in the MVE group or BMF group than in controls since a 4-week exposure (*p* < 0.05 or *p* < 0.01, Fig. 2c). Moreover, a significant increase in total leukocyte counts were observed both in the BMF group and MVE group (*p* < 0.05 or *p* < 0.01, Fig. 2d). The total leukocyte counts started to increase greatly since a 4-week exposure and remained elevated to the 24 weeks of exposure. Differential cell counts showing that increase in total leukocyte counts were due mainly to increases in alveolar macrophages and neutrophils counts (*p* < 0.05 or *p* < 0.01, Fig. 2e-f), and the alveolar macrophages have the highest percentage in total leukocyte counts.

**Fig. 3:**
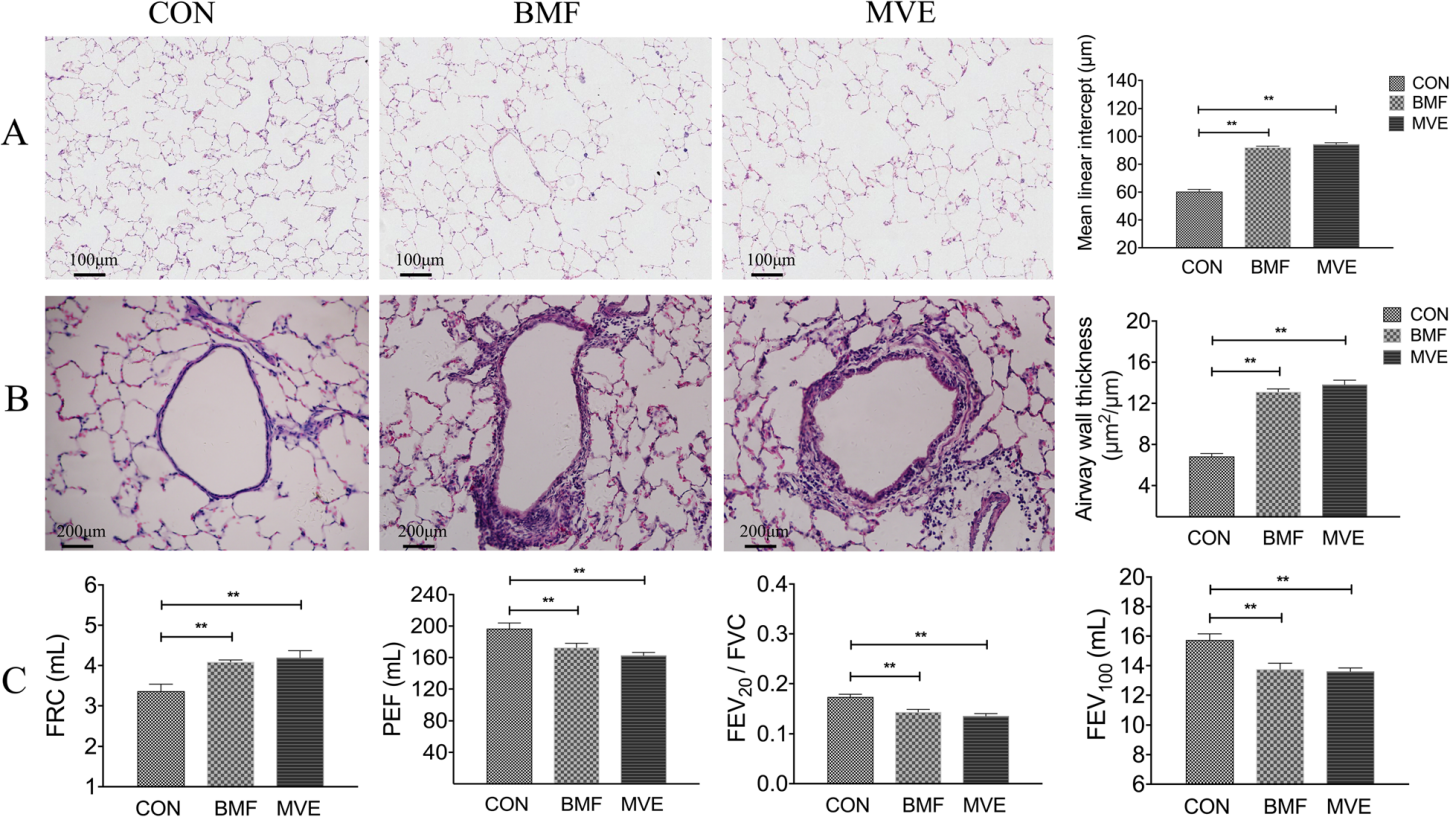
Exposure to biomass fuel (BMF) and motor vehicle exhaust (MVE) induces effects consistent with chronic obstructive pulmonary disease in rats. **a** Lung sections show significant increases in the mean linear intercept after 24 weeks of BMF and MVE exposure. **b** The thickness of the small airway wall increased significantly in the rats after 24 weeks of BMF and MVE exposure. **c** Effects of BMF and MVE exposure on rat pulmonary function test results. *n* = 6. **p < 0.01. CON, control group

Exposure to PM also induced COPD-like changes in rat lung. Histological analysis showed that significant increases in the mean linear intercept after 24 weeks of exposure in both the BMF (*p* < 0.01) and MVE (*p* < 0.01) groups compared to controls (Fig. 3a). Long-term PM exposure damaged the lung parenchyma. After 24 weeks BMF and MVE particles exposure, the thickness of bronchial walls in the lungs of rats was increase greatly to that of controls (*p* < 0.01, Fig. 3b), suggesting that the PM exposures induced airway remodeling. Moreover, after 24-weeks of exposure, the pulmonary function test results of FRC were significantly increased, but the PEF, FEV_20_/FVC and FEV_100_ were decreased significantly both in the BMF group and MVE group than from controls (*p* < 0.01, Fig. 3c). Taken together, these findings suggest that chronic exposure to BMF and MVE induced COPD-like changes in the rat lung.

### Effect of PM exposure on the gut and lung microbiota

To determine whether chronic exposure to ambient PM induces intestinal and lung microbial shifts, we performed 16S rRNA gene sequencing of proximal colon contents and BALF samples from rats exposed to BMF or MVE after 4, 12, and 24 weeks of exposure.

**Fig. 4:**
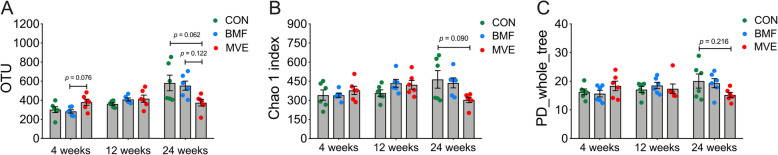
Gut microbial abundance and diversity following exposure to biomass fuel or motor vehicle exhaust. Comparison of the operational taxonomic units (OTUs) (**a**) and alpha diversity (as assessed by the Chao 1 (**b**) and PD_whole_tree (**c**) indices; *n* = 6 per group). CON, control group; BMF, biomass fuel exposure group; MVE, motor vehicle exhaust exposure group

**Fig. 5:**
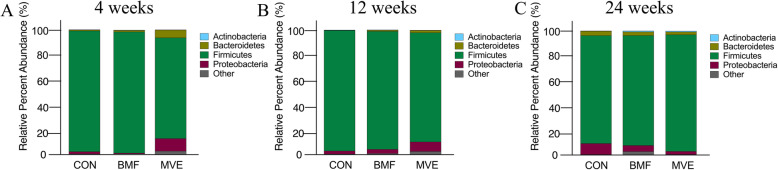
Relative proportions of major bacterial phyla following exposure to particulate matter. Microbial abundance was measured in colon contents from rats exposed to clean air (CON) and to particulate matter from biomass fuel (BMF) or motor vehicle exhaust (MVE)

For gut microbial analysis, after filtering for low-quality reads, 4,479,175 sequence reads from 54 samples were used for analysis and resulted in 22,000 operational taxonomic units (OTU). Comparisons between the three groups showed that intra-individual diversity, as measured by the number of OTUs, decreased significantly in the MVE group after 24 weeks of exposure (*p* = 0.062, vs CON group; *p* = 0.122, vs BMF group, Fig. 4a). Other indices (Chao 1and PD_whole_tree) were calculated to estimate the within-sample (alpha) diversity. The Chao 1 (*p* = 0.090, vs CON group; Fig. 4b) and PD_whole_tree indices (*p* = 0.216, vs CON group, Fig. 4c) were lower in the MVE group after 24 weeks exposure. The reduced richness of OTUs and alpha diversity in the gut microbiota suggest a possible deficiency in healthy microflora in the BMF and MVE groups. To investigate the difference between gut microbial communities in the three groups, we analyzed the taxonomical community structure of the microbiome in colon contents. At the phylum level, all samples at all measurement time points from the three groups contained four major bacterial phyla: Bacteroidetes, Firmicutes, Proteobacteria, and Actinobacteria. The first three phyla accounted for over 97% of the total sequences in all three groups (Fig. 5). After a 4-week exposure, the MVE group had a lower relative abundance of Firmicutes (*p* = 0.003 vs. the control group; *p* = 0.002 vs. the BMF group) but a higher relative abundance of Proteobacteria (*p* = 0.099 vs. the control group; *p* = 0.055 vs. the BMF group, Fig. 5a). After a 12-week exposure, the MVE group exhibited relative abundances of Firmicutes (*p* = 0.019 vs. the control group; *p* = 0.072 vs. the BMF group, Fig. 5b) that were consistent with the trend seen after 4 weeks of exposure. However, after 24 weeks of exposure, the three groups exhibited a similar relative abundance of four major bacterial phyla (*p* > 0.05, Fig. 5c). These findings indicate gut microbiota dysbiosis after PM exposure.

**Fig. 6:**
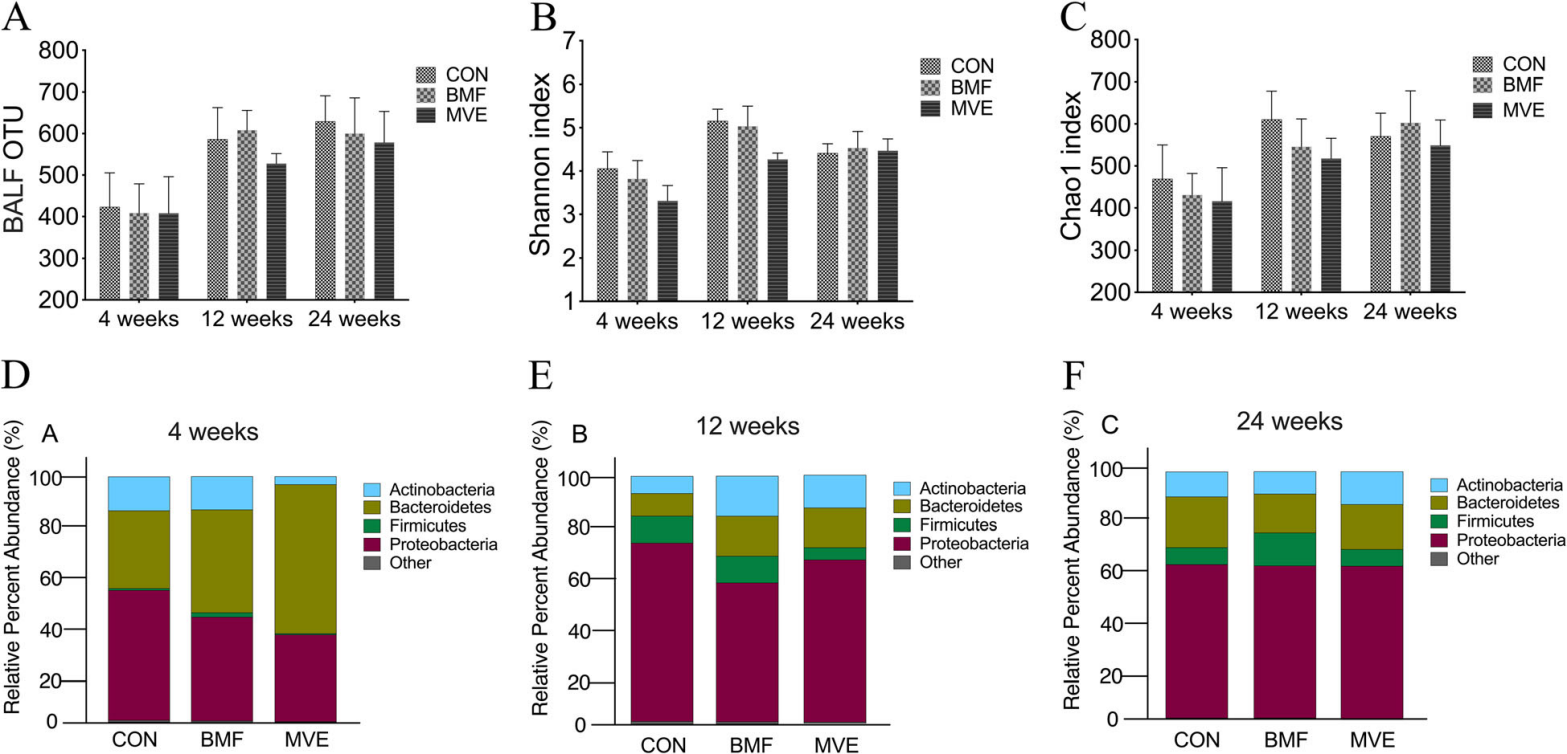
Effect of particulate matter exposure on community diversity and richness of the lung microbiota. Operational taxonomic units (OTUs) (**a**) and microbial diversity (Shannon index (**b**) and Chao 1 index (**c**)). Composition and relative abundances of bacterial phyla in different groups after 4, 12 and 24 weeks exposure (**d**-**f**). Results are expressed as mean ± SD; *n* = 6 rat. CON, control group; BMF, biomass fuel exposure group; MVE, motor vehicle exhaust exposure group

For lung microbial analysis, high-quality sequence reads were used for subsequent analyses and resulted in 28,336 OTUs. As shown in Fig. 6a, there were no significant difference of number of OTUs between the three groups in 4, 12, and 24 weeks time-point (*p* > 0.05). Chao 1 and the Shannon index indicated that there was no significant difference between the three groups in 4, 12, and 24 weeks time-point (*p* > 0.05, Fig. 6b-c). At the phylum level, all samples from the control group, BMF group and MVE group also contained the four major bacterial phyla: Bacteroidetes, Firmicutes, Proteobacteria, and Actinobacteria. Despite the control group, BMF group and MVE group showed different microbial composition in 4 and 12 weeks, there was no statistically significant difference with its limited sample size (*p* > 0.05, Fig. 6d-f).

### Quantification of microbial metabolites

**Fig. 7:**
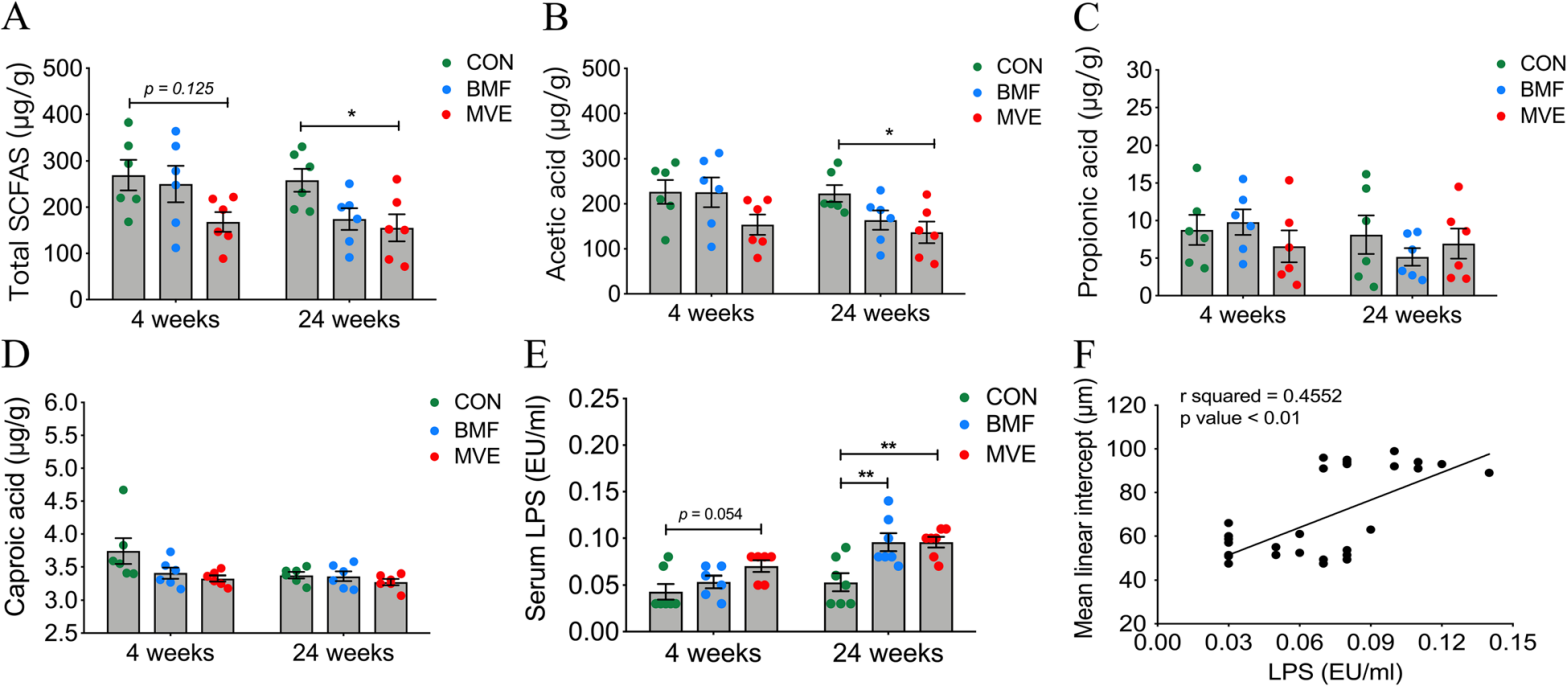
Microbial metabolites in colon contents of controls (CON) and rats exposed to biomass fuel (BMF) or motor vehicle exhaust (MVE). **a** Levels of total short-chain fatty acids (SCFAs) were lower in the MVE groups after 24 weeks of exposure. **b** Levels of acetic acid were lower in the MVE groups, especially after 24 weeks of exposure. **c**, **d** Levels of propionic acid and caproic acid did not differ between groups. **e** Serum lipopolysaccharide (LPS) levels in the three groups after 4 and 24 weeks of exposure. **f** Elevated serum LPS levels were correlated with the mean linear intercept. Boxes and the inside line represent the mean ± SD; each dot corresponds to a sample. *n* = 6. *p < 0.05, **p < 0.01

We analyzed the levels of SCFAs in colon contents following 4- and 24-week exposures using gas chromatography. Acetic, propionic, and caproic acids were detected in all colon contents, but butyric, isobutyric, valeric, and isovaleric acids were not detected in some samples. Levels of total SCFAs were significantly lower in the MVE group than in controls (*p* = 0.125) after 4 weeks and 24-week exposure (*p* = 0.041, Fig. 7a). Moreover, a significant decrease in acetic acid was observed in colon contents from the MVE group relative to contents in controls (*p* = 0.036, Fig. 7b). Propionic and caproic acids exhibited similar levels after PM exposure (Fig. 7c, d).

Serum LPS levels were higher in the MVE group than in controls after a 4-week exposure (*p* = 0.054). After a 24-week exposure, serum LPS levels were significantly higher in both the BMF (*p* = 0.007) and MVE (*p* = 0.007) groups than in controls (Fig. 7e), and exhibited a weak but significant positive correlation with the pulmonary mean linear intercept (*r*^2^ = 0.4552, *p* < 0.001; Fig. 7f).

## Discussion

In previous work, we found that elevated PM concentrations were associated with increased COPD prevalence and diminished respiratory function [4]. Moreover, use of clean fuels and improved ventilation have been associated with better pulmonary function and a reduced risk of COPD [5]. We then established a rat COPD model using exposure to biomass fuel and motor vehicle exhaust to study the pathophysiology of PM-induced COPD [6], but the underlying biological mechanisms are still unclear. PM research has mostly focused on the lung inflammatory response to inhalation, endothelial dysfunction, and oxidative stress, but the effects of PM on the microbiota and its role in the pathogenesis of COPD are largely unknown. In the present study, we demonstrated that chronic exposure to PM induced COPD-like characteristic pathological changes and pulmonary inflammation in rats that were concomitant with a decrease in the abundance and diversity of gut microbiota. Moreover, the decreased SCFA levels, increased LPS concentration and significant COPD-like changes were observed at the same time-points.

Recent studies have suggested a role for the gut microbiota in the development of respiratory diseases [21], although the connection between the lung and the gut is poorly understood. In the present study, we found that PM exposure was associated with decreased abundance and diversity in bacterial populations, similar to the effects reported for fine PM on diabetes and obesity [22–24]. These data from epidemiological and animal studies suggest that long-term exposure to PM causes gut microbiota dysbiosis and may subsequently contribute to increased risk of diabetes and obesity. Importantly, a chronic exposure (24 weeks) to PM reduced OTUs and alpha diversity, consistent with the findings of a study conducted in mice [22]. In addition to diversity, bacterial relative abundance was also affected, and may partially mediate the association of PM with COPD. Notably, the abundance of Firmicutes was first reduced in rats exposed to MVE group, but after 24 weeks of exposure, the relative abundance of Firmicutes increased from 80 to 97%, at the same time that the relative abundance of Firmicutes in rats exposed to BMF decreased from 97 to 88%. Our findings show that gut microbiota dysbiosis in a PM-induced rat model of COPD may depend on exposure duration, the dose, or the sources of PM.

The imbalance in gut microbiota leads to the migration of bacteria or bacterial products, such as LPS, from the intestinal lumen to mesenteric lymph nodes or the circulation, in association with an inflammatory response [25, 26]. LPS causes the production of inflammatory mediators, and the process can result in pathological changes characteristic of COPD in the lung [27]. Certainly, the damage to the airway epithelial cells and lung tissues might enable microbial endotoxin to enter the circulation via the pulmonary route. However, previous research confirmed pro-inflammatory effects observed from PM are driven largely by the insoluble components of the PM mixture, and are not caused by endotoxin [28]. Furthermore, we also tested the concentration of endotoxin from DMSO-extract of particulate matter, and the data suggest that the concentration of endotoxin from China fir sawdust, gasoline-powered motorcycle and the clean air was not significantly different. Therefore, the increase level of LPS in present study might be most likely come from the gastrointestinal tract.

It should be noted that after exposure for 24 weeks, the lung microbial from the three groups showed similar microbial diversity and composition in rat lung. The result in this study is somewhat different to previous study looking at microbiota changes in murine models using fine particulate matter (PM2.5) exposure [29]. The study by Li and colleagues found that PM2.5 exposure significantly altered the richness, evenness, and composition of the lung microbiota in mice. Several experimental factors may explain the discordant findings. Different source of PM (e.g. PM2.5, used in the study by Li, was purchased from the standard reference materials), the animal species and strain used (mice vs rat), and the duration and intensity of the exposure (mice were exposed to PM2.5 by intratracheal instillation for a total of 7 day).

SCFAs are metabolized by the gut microbiota from otherwise indigestible fiber-rich foods and are involved in metabolism. SCFAs have anti-inflammatory properties, are a source of energy for colonocytes, improve gut barrier function, and reduce intestinal bacterial translocation [30, 31]. A high-fiber diet has been suggested to decrease COPD risk, primarily in current and former smokers [32, 33]. Animal experiments also support this SCFA-related mechanism; an increase in dietary SCFAs was reported to attenuate the development of smoking-related emphysema in rats and mice [34, 35]. Our findings showed that the SCFA levels were decreased after 24-weeks exposure, and we also observed COPD-like changes at the same time-points. However, further research is required to establish whether the decrease in SCFA play any causative role in the COPD-like structural or functional changes in this model.

Although the study demonstrates a notable impact of inhaled PM on the gut microbiota and implicates it in the development of COPD, several limitations should be noted. First, in the absence of interventional research, it is difficult to ascertain whether gut microbiota dysbiosis is a cause or a consequence of COPD. Second, although exposure to PM was shown to alter the composition and function of gut microbiota, how PM inhalation affects the gut microbiota remains to be determined. Third, steroid insensitivity has been commonly found in COPD patients and animal models. The effect of a steroid in our rat COPD model is of clinical importance and needs further investigation. Finally, we cannot yet ascertain whether PM induction of gut microbiota dysbiosis is time- or dose-dependent. More studies are warranted to better understand the pathogenesis of gut microbiota dysbiosis after chronic exposure to ambient PM.

## Conclusions

Chronic exposure to ambient PM decreases the levels of SCFAs in the colon and induces gut microbial shifts and translocation in a rat COPD model.

## Supplementary information

**Additional file 1: Supplementary Table 1.** Concentrations of particulate matter (PM) and gaseous pollutants measured during exposure. **Supplementary Table 2.** The elemental composition of PAHs in ambient BMF and MVE samples. **Supplementary Table 3.** The elemental composition of OC/EC in ambient BMF and MVE samples. **Supplementary Table 4.** The metal composition in ambient BMF and MVE samples. **Supplementary Table 5.** The concentration of endotoxin in DMSO-extract of particulate matter.

## Data Availability

Please contact author for data requests.

## Abbreviations

BALF: Bronchoalveolar lavage fluid
BMF: Biomass fuel
COPD: Chronic obstructive pulmonary disease
FEV20: Forced expiratory volume in 20 s
FEV100: Forced expiratory volume in 100 s
FRC: Functional residual capacity
LPS: Lipopolysaccharide
MVE: Motor vehicle exhaust
PEF: Peak expiratory flow
PM: Particulate matter
SCFA: Short-chain fatty acid

## References

[1] Liu W, Huang C, Hu Y (2016). Associations of gestational and early life exposures to ambient air pollution with childhood respiratory diseases in Shanghai, China: a retrospective cohort study. Environ Int.

[2] Guan WJ, Zheng XY, Chung KF (2016). Impact of air pollution on the burden of chronic respiratory diseases in China: time for urgent action. Lancet.

[3] Zeki AA, Flayer CH, Haczku A (2019). A burning need to redefine airways disease: biomass smoke exposure identified as a unique risk factor for asthma-chronic obstructive pulmonary disease overlap in low-and middle-income countries. J Allergy Clin Immunol.

[4] Liu S, Zhou Y, Liu S (2016). Association between exposure to ambient particulate matter and chronic obstructive pulmonary disease: results from a cross-sectional study in China. Thorax.

[5] Zhou Y, Zou Y, Li X (2014). Lung function and incidence of chronic obstructive pulmonary disease after improved cooking fuels and kitchen ventilation: a 9-year prospective cohort study. PLoS Med.

[6] He F, Liao B, Pu J (2017). Exposure to ambient particulate matter induced COPD in a rat model and a description of the underlying mechanism. Sci Rep.

[7] Salim SY, Kaplan GG, Madsen KL (2014). Air pollution effects on the gut microbiota: a link between exposure and inflammatory disease. Gut Microbes.

[8] Yu G, Gail MH, Consonni D (2016). Characterizing human lung tissue microbiota and its relationship to epidemiological and clinical features. Genome Biol.

[9] Wang Z, Singh R, Miller BE (2018). Sputum microbiome temporal variability and dysbiosis in chronic obstructive pulmonary disease exacerbations: an analysis of the COPDMAP study. Thorax.

[10] Bouquet J, Tabor DE, Silver JS (2020). Microbial burden and viral exacerbations in a longitudinal multicenter COPD cohort. Respir Res.

[11] Xu X, Wang X, Hu Y (2020). Short-term effects of thinning on the development and communities of understory vegetation of Chinese fir plantations in Southeastern China. PeerJ.

[12] Wang X, Chen M, Zhong M (2017). Exposure to concentrated ambient PM2.5 shortens lifespan and induces inflammation- associated signaling and oxidative stress in drosophila. Toxicol Sci.

[13] Wu SP, Tao S, Zhang ZH (2005). Distribution of particle-phase hydrocarbons, PAHs and OCPs in Tianjin, China. Atmos Environ.

[14] Li N, He F, Liao B (2017). Exposure to ambient particulate matter alters the microbial composition and induces immune changes in rat lung. Respir Res.

[15] Hsia CC, Hyde DM, Ochs M (2010). An official research policy statement of the American Thoracic Society/European Respiratory Society: standards for quantitative assessment of lung structure. Am J Respir Crit Care Med.

[16] 16S metagenomic sequencing library preparation. San Diego: Illumina. Updated 2014. Retrieved July 15, 2016, from https://www.illumina.com/.

[17] Klindworth A, Pruesse E, Schweer T (2013). Evaluation of general 16S ribosomal RNA gene PCR primers for classical and next-generation sequencing-based diversity studies. Nucleic Acids Res.

[18] Rocafort M, Noguera-Julian M, Rivera J (2019). Evolution of the gut microbiome following acute HIV-1 infection. Microbiome.

[19] Liu S, Zhou Y, Wang X (2007). Biomass fuels are the probable risk factor for chronic obstructive pulmonary disease in rural South China. Thorax.

[20] Wang J (2013). China air quality online monitoring and analysis platform.

[21] Fujimura KE, Lynch SV (2015). Microbiota in allergy and asthma and the emerging relationship with the gut microbiome. Cell Host Microbe.

[22] Wang W, Zhou J, Chen M (2018). Exposure to concentrated ambient PM2.5 alters the composition of gut microbiota in a murine model. Part Fibre Toxicol.

[23] Alderete TL, Jones RB, Chen Z (2018). Exposure to traffic-related air pollution and the composition of the gut microbiota in overweight and obese adolescents. Environ Res.

[24] Liu T, Chen X, Xu Y (2019). Gut microbiota partially mediates the effects of fine particulate matter on type 2 diabetes: evidence from a population-based epidemiological study. Environ Int.

[25] Cani PD, Jordan BF (2018). Gut microbiota-mediated inflammation in obesity: a link with gastrointestinal cancer. Nat Rev Gastroenterol Hepatol.

[26] Cox AJ, West NP, Cripps AW (2015). Obesity, inflammation, and the gut microbiota. Lancet Diabetes Endocrinol.

[27] Korsgren M, Linden M, Entwistle N (2012). Inhalation of LPS induces inflammatory airway responses mimicking characteristics of chronic obstructive pulmonary disease. Clin Physiol Funct Imaging.

[28] Wegesser TC, Last JA (2008). Lung response to coarse PM: bioassay in mice. Toxicol Appl Pharmacol.

[29] Li J, Hu Y, Liu L (2020). PM2.5 exposure perturbs lung microbiome and its metabolic profile in mice. Sci Total Environ.

[30] Lau WL, Vaziri ND (2019). Gut microbial short-chain fatty acids and the risk of diabetes. Nat Rev Nephrol.

[31] Sanna S, van Zuydam NR, Mahajan A (2019). Causal relationships between gut microbiome, short-chain fatty acids and metabolic diseases. Nat Genet.

[32] Kaluza J, Harris H, Wallin A (2018). Dietary fiber intake and risk of chronic obstructive pulmonary disease: a prospective cohort study of men. Epidemiology.

[33] Varraso R, Chiuve SE, Fung TT (2015). Alternate healthy eating index 2010 and risk of chronic obstructive pulmonary disease among US women and men: prospective study. BMJ.

[34] Tomoda K, Kubo K, Yamamoto Y (2014). Alteration in gut environment accelerates emphysematous lesions by cigarette smoke in rats discontinuously fed with a fiber-free diet. Am J Respir Crit Care Med.

[35] Tomoda K, Kubo K, Dairiki K (2015). Whey peptide-based enteral diet attenuated elastase-induced emphysema with increase in short chain fatty acids in mice. BMC Pulm Med.

