# Supplementary material for: Chronic exposure to ambient particulate matter induces gut microbial dysbiosis in a rat COPD model
Source: Respir Res. 2020 Oct 19;21:271. doi: 10.1186/s12931-020-01529-3 (PMC7574574; doi:10.1186/s12931-020-01529-3)
Supplement: Supplementary file 1 — Additional file 1: Supplementary Table 1. Concentrations of particulate matter (PM) and gaseous pollutants measured during exposure. Supplementary Table 2. The elemental composition of PAHs in ambient BMF and MVE samples. Supplementary Table 3. The elemental composition of OC/EC in ambient BMF and MVE samples. Supplementary Table 4. The metal composition in ambient BMF and MVE samples. Supplementary Table 5. The concentration of endotoxin in DMSO-extract of particulate matter. [file 12931_2020_1529_MOESM1_ESM.docx]

**Supplementary Table**

**Table 1. Concentrations of particulate matter (PM) and gaseous pollutants measured during exposure.**

| **Test items** | **CON** | **BMF** | **MVE** |
| --- | --- | --- | --- |
| PM10 (mg/m^3^) | — | 25.87±2.99 | 1.86±0.20 |
| PM2.5 (mg/m^3^) | — | 21.91±1.84 | 1.85±0.24 |
| PM1 (mg/m^3^) | — | 19.60±1.76 | 1.82±0.27 |
| NO_1_ (ppm) | — | 0.78 ± 0.10 | 0.31 ± 0.07 |
| NO_X_ (ppm) | — | 0.78 ± 0.10 | 0.31 ± 0.07 |
| SO_2_ (ppm) | — | — | 0.82 ± 0.10 |
| CO (ppm) | — | 110.1±15.4 | 129.0±12.6 |
| O_2_ (%) | 20.9 ± 0.04 | 20.7±0.06 | 20.4±0.05 |
| Humidity (%) | 60.1±5.6 | 66.0±9.3 | 73.5±9.9 |
| Temperature (℃) | 24.9±0.5 | 26.1±1.8 | 27.5±4.4 |

Values are shown as mean ± SD. CON, control group; BMF, biomass fuel exposure group; MVE, motor vehicle exhaust exposure group.

**Table 2. The elemental composition of PAHs in ambient BMF and MVE samples.**

| **PAHs** | **BMF (mg/kg)** | **MVE (mg/kg)** |
| --- | --- | --- |
| Naphthalene | 6.95 ± 2.24 | — |
| Acenaphthylene | — | 0.03 ± 0.03 |
| Acenaphthene | — | — |
| Fluorene | — | 0.06 ± 0.05 |
| Phenanthrene | 2.34 ± 1.07 | 2.22 ± 1.30 |
| Anthrancene | 0.20 ± 0.05 | 0.07 ± 0.03 |
| Fluoranthene | 1.04 ± 0.28 | 4.17 ± 3.16 |
| Pyrene | 0.16 ± 0.13 | 7.71 ± 5.80 |
| Benzo anthracene | 0.22 ± 0.19 | 2.25 ± 1.24 |
| Chrysene | — | 1.05 ± 0.73 |
| Benzo[b+j] fluoranthene | — | 4.02 ± 3.90 |
| Benzo[k] fluoranthene | — | 0.59 ± 0.38 |
| Benzo[a] pyrene | — | 2.19 ± 1.70 |
| Benzo[e] pyrene | — | 2.69 ± 1.30 |
| Dibenz[a,h] anthracene | — | — |
| Benzo[g,h,i] perylene | — | — |
| Indeno[1,2,3-cd] pyrene | — | 4.81 ± 2.75 |

Results are expressed as mean ± SD; Concentrations were expressed as mg/kg; PAHs, Polycyclic Aromatic Hydrocarbons; Horizontal line mean below detection limit; BMF, biomass fuel exposure group; MVE, motor vehicle exhaust exposure group.

**Table 3. The elemental composition of OC/EC in ambient BMF and MVE samples.**

| **OC/EC** | **BMF (ng/μg)** | **MVE (ng/μg)** |
| --- | --- | --- |
| TC | 117.24 ± 3.39 | 153 ± 21.66 |
| OC | 116.70 ± 3.31 | 146.11 ± 19.79 |
| EC | 0.54 ± 0.08 | 6.89 ±1.87 |
| OC/EC | 28.10 ± 1.98 | 10.72 ± 2.11 |
| OC1 | 67.33 ± 0.63 | 36.72 ± 7.67 |
| OC2 | 26.50 ± 1.13 | 102.09 ± 11.62 |
| OC3 | 6.86 ± 0.37 | 4.97 ± 0.38 |
| OC4 | 1.03 ± 0.18 | 1.36 ± 0.46 |
| EC1 | 15.4 ± 1.05 | 7.79 ±1.48 |
| EC2 | 0.08 ± 0.02 | 0.08 ± 0.04 |
| EC3 | 0.05 ± 0.02 | — |
| OP2 | 14.99 ± 1.02 | 0.97 ±0.34 |

Results are expressed as mean ± SD; OC/EC, organic carbon/elemental carbon; Horizontal line mean below detection limit; BMF, biomass fuel exposure group; MVE, motor vehicle exhaust exposure group.

**Table 4. The metal composition in ambient BMF and MVE samples.**

| **Metal** | **BMF (ng/μg)** | **MVE (ng/μg)** |
| --- | --- | --- |
| Na | 0.003 ±0.003 | 0.16 ± 0.09 |
| Mg | 0.01 ±0.01 | 0.11 ± 0.11 |
| Al | — | — |
| Si | — | 0.04 ± 0.03 |
| S | 0.15 ± 0.10 | 1.13 ± 0.26 |
| Cl | 0.20 ± 0.15 | 0.03 ± 0.03 |
| K | 0.002 ± 0.001 | 0.03 ± 0.01 |
| Ca | — | 0.52 ± 0.13 |
| Ti | — | 0.002 ± 0.002 |
| V | — | — |
| Cr | — | — |
| Mn | 0.001 ± 0.001 | 0.002 ± 0.001 |
| Fe | — | 0.27 ± 0.10 |
| Co | — | — |
| Ni | — | — |
| Cu | — | 0.01 ± 0.01 |
| Zn | — | 0.30 ± 0.14 |
| Rb | — | — |
| Sb | 0.002 ± 0.001 | — |
| Ba | 0.004 ± 0.002 | 0.02 ± 0.01 |
| Pb | — | 0.01 ± 0.01 |

Results are expressed as mean ± SD; Horizontal line mean below detection limit; BMF, biomass fuel exposure group; MVE, motor vehicle exhaust exposure group.

**Table 5. The concentration of endotoxin in DMSO-extract of particulate matter.**

| **Sample No.** | **Source of PM** | **Test result (EU/ml)** |
| --- | --- | --- |
| 1 | Traffic-related PM | 99.20 |
| 2 | PM generated during cooking | 47.60 |
| 3 | Outdoor atmosphere | 25.60 |
| 4 | BMF produced by China fir sawdust | 12.0 |
| 5 | MVE produced by gasoline-powered motorcycle | 10.0 |
| 6 | Clean air from specific pathogen-free room | 12.5 |

BMF, biomass fuel exposure group; MVE, motor vehicle exhaust exposure group; PM, particulate matter.
